# Oral manifestations of sporotrichosis: A neglected disease

**DOI:** 10.4317/jced.59040

**Published:** 2023-01-01

**Authors:** Aline-Corrêa Abrahão, Michelle Agostini, Tamires-Rocha de Oliveira, Cesar-Werneck Noce, Arley-Silva Júnior, Márcia-Grillo Cabral, Mário-José Romañach, Sandra-Regina Torres

**Affiliations:** 1 DDS, PhD. Department of Oral Diagnosis and Pathology, School of Dentistry, Federal University of Rio de Janeiro (UFRJ), Rio de Janeiro, Brazil; 2 DDS, PhD. Dental Service, Clementino Fraga Filho Univeristy Hospital (HUCFF), Federal University of Rio de Janeiro, Rio de Janeiro, Brazil; 3 DDS. Dental Service, Clementino Fraga Filho Univeristy Hospital (HUCFF), Federal University of Rio de Janeiro, Rio de Janeiro, Brazil

## Abstract

Sporotrichosis is an uncommon subacute or chronic infection caused by Sporothrix spp. In some urban areas of Latin America, sporotrichosis has been considered an emergent cosmopolitan disease of zoonotic transmission by domestic cats. There are four different clinical forms of the disease: fixed cutaneous, lymphocutaneous, multifocal or disseminated cutaneous, and extracutaneous. The oral mucosa is rarely involved, usually as unspecified chronic ulcers in the context of multifocal or disseminated cutaneous form of systemic sporotrichosis. Microscopical features include chronic granulomatous inflammation containing microabscesses and fungal hyphae positive for Periodic acid Schiff and silver-based stains. The diagnosis of sporotrichosis is usually based on culture detection and strict correlation of clinical, microscopical and laboratorial data. We herein contribute with two additional illustrative cases of oral manifestation of sporotrichosis in immunocompromised patients from an endemic urban area from Rio de Janeiro-Brazil.

** Key words:**Sporotrichosis, ulcer, oral cavity, immunosuppression.

## Introduction

Sporotrichosis is an uncommon subacute or chronic infection caused by the thermodimorphic fungi *Sporothrix spp.* that usually affects patients from tropical and subtropical regions (1-4). Four different clinical forms of the disease are recognized: fixed cutaneous, lymphocutaneous, multifocal or disseminated cutaneous, and extracutaneous (5). The oral mucosa involvement is characterized by unspecified chronic ulcers in the context of multifocal or disseminated cutaneous form of systemic sporotrichosis, usually in immunocompromised patients (1,6-8).

Microscopically, chronic granulomatous inflammation containing microabscesses and eosinophilic material of radiate, star-like, or asteroid conFigurations around the fungal hyphae (Splendore-Hoeppli phenomenon) is helpful in making a presumptive diagnosis of sporotrichosis. Periodic acid Schiff and silver-based stains may aid in the identification of Sporothrix spp., but the definitive diagnosis is based on culture detection and strict correlation of clinical, microscopical and laboratorial data (2,9).

The exact prevalence of sporotrichosis worldwide is not well established since it is not a reporTable disease. The cases reported in the literature are frequently from patients from the United States, South America (Brazil, Colombia, Guatemala, Mexico, Peru), Asia (China, India, Japan), and Australia, being rarely reported in Europe (10). In the last decades, sporotrichosis has been considered an emergent cosmopolitan disease of zoonotic transmission by domestic cats, being the most frequent subcutaneous mycosis in Latin America (3), mainly in Brazil, with zoonotic outbreaks being reported in endemic areas from São Paulo, Rio Grande do Sul and the northeast region (3,11). We contribute with two additional illustrative cases of oral manifestations of sporotrichosis in patients from an endemic urban area from Rio de Janeiro, where approximately 5000 cases of human sporotrichosis caused by *Sporothrix brasiliensis* were diagnosed up to 2015 (2-4,7,12).

## Case Report

**Figure 1:**
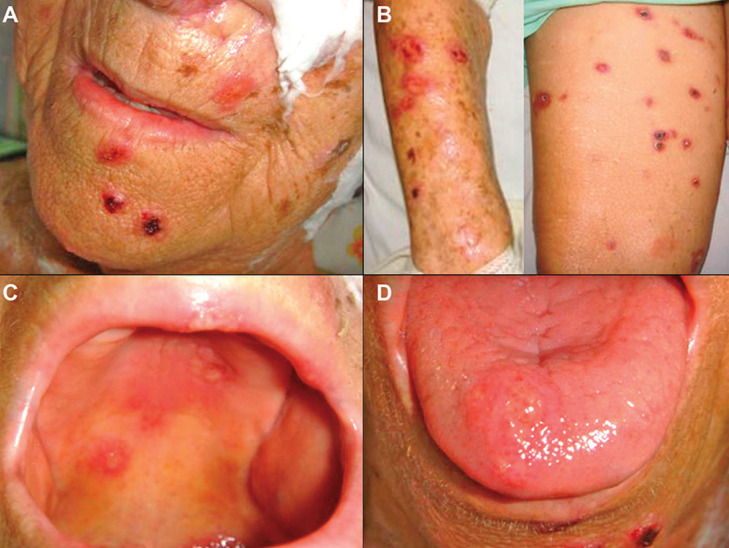
Clinical features of disseminated sporotrichosis with oral manifestation (case 1). (A,B) Multiple cutaneous, erosive, and erythematous papules with crust formation on perioral region, arms and thigh. (C,D) Multiple ulcers of varying size in the palate and dorsal surface of the anterior tongue.

Case 1 was an 80-year-old female referred for evaluation of painful oral ulcers. Medical history included fever and cough for the last 2 months, and tuberculosis confirmed by culture of *Mycobacterium tuberculosis*, with discontinued treatment with rifampicin and isoniazid. Physical examination revealed multiple cutaneous erosive, and erythematous papules with crust formation on the face, an irregular large ulcer on the left breast, an erythematous plaque with yellowish papules on the right arm and necrotic ulcers with crusts on her 3rd left toe. Intra-oral examination revealed multiple ulcers and erythematous plaques in the hard and soft palates, as well as on the dorsum and lateral surfaces of the tongue (Fig. 1). The patient also reported dysphagia, dysphonia, and chronic nasal obstruction, and of note, the death of her cat few months earlier, presenting lesions on the skin.

**Figure 2:**
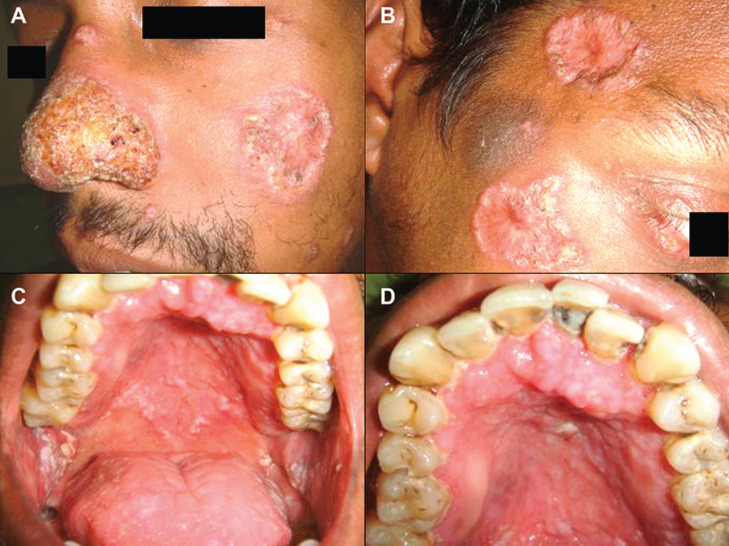
Clinical features of disseminated sporotrichosis with oral manifestation in a HIV patient (case 2). A and B. Multiple crateriform ulcers affecting the skin of the centrofacial region. C and D. Diffuse ulcerated mucosa lesions with granular surface in the hard palate and gingivae, associated with pseudomembranous candidiasis co-infection in tongue, buccal mucosa and palate.

Case 2 was a 31-year-old HIV-positive male referred for evaluation of multiple cutaneous ulcers. The patient was under anti-retroviral therapy (efavirenz, stavudine and lamivudine), with CD4 count of 114 cells/mm3 and viral load of 76,023 copies/ml. He also reported use of tobacco and alcohol. Upon physical examination, multiple cutaneous ulcers with elevated margins and crust formation were observed on the face, chest, and upper limbs. Intra-oral examination showed diffuse superficial granular ulcers of moriform surface and variable size, located in the hard/soft palate, gingivae, buccal mucosa and tongue (Fig. 2). White patchy lesions were also noted in both lateral borders the tongue, as well as white removable plaques with erythematous background in the dorsum of the tongue, buccal mucosa, palate, and labial mucosa. Ulcerative lesions were also identified on the esophagus.

**Figure 3:**
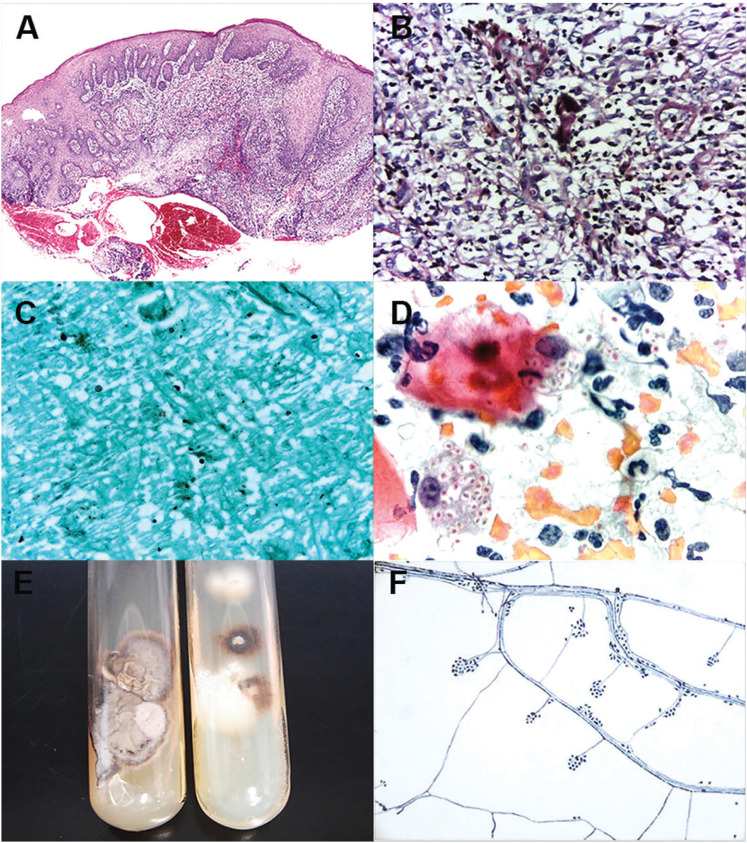
Microscopic features of oral sporotrichosis. A. Fragment of oral mucosa presenting granulomatous chronic inflammation with multinucleated giant cells, and pseudoepitheliomatous hyperplasia of the overlying epithelium. Fungi structures were highlighted by (B) Periodic acid Schiff and (C) Gomori-Grocott metenamine silver stain, and also observed in the smears collected by (D) exfoliative cytology (Papanicolaou). E and F. Yeast culture of sporotrichosis. Membranous colonies of white-creamy and black-leathery color (E). Microscopic evaluation of the colonies showing septate hyphae with “daisy-like” arrangement at the end of the conidiophores (F).

In both cases, incisional biopsies of cutaneous and oral lesions were performed. Histological evaluation showed ulcerated fragments containing diffuse chronic granulomatous inflammation located deeply in the connective tissue. Periodic acid Schiff and Grocott-Gomori methenamine-silver stains highlighted round yeast-like fungal structures, which showed spherical, oval, or elongated (cigar-shaped) morphology by exfoliative cytology evaluation (Fig. 3). Yeast culture showed at first, white-creamy membranous colonies which later turned to black-leathery color. Microscopic evaluation of the colonies showed septate hyphae of pyriform conidia with typical “daisy-like” arrangement at the end of the conidiophores (Fig. 3). The microscopic diagnosis was of sporotrichosis in both cases. The correlation between clinical and microscopic findings of both cases lead to the final diagnosis of disseminated sporotrichosis. The second patient was then diagnosed with acquired immunodeficiency syndrome (AIDS) due to CD4 count and viral load associated with deep fungal infection.

The first patient was submitted to amphotericin B treatment for 15 days (1 mg/kg daily), which was subsequently changed to 400 mg of itraconazole, daily, for 2 weeks. As cutaneous lesions persisted, patient was submitted to itraconazole 800mg daily for 6 months. Therapy for tuberculosis followed with ethambutol and isoniazid. Clinical improvement was observed for both cutaneous and oral lesions after 6 months, but the patient did not attend the following appointments. The second patient received 1.5 mg/kg of amphotericin B for 2 weeks, followed by 400 mg of itraconazole, daily, for the next 2 weeks. A good response was observed, with complete remission of cutaneous and oral lesions; the patient missed long-term follow up appointments.

## Discussion

Sporotrichosis was first described in 1898 by Benjamin Schenck in the United States of America. The fungus isolated from the cutaneous lesions of a patient treated by Dr. Schenck was identified by the pathologist Erwin F. Smith as belonging to the genus *Sporotrichum*, which was designated as *Sporothrix schenckii* by Hektoen and Perkins in 1900. Although *S. schenckii* had been considered the only species responsible for sporotrichosis during many years, it is currently known as a group of phylogenetically related species that comprises 51 taxons, divided into clinical and environmental clades. *S. schenckii* and *Spotpthrix globosa* have already been isolated from humans, animals, and soil, while *Sporothrix brasiliensis* is frequently isolated from human and feline clinical samples. The infection of sporotrichosis (formerly known as “Rosebush” or “Gardener’s” mycosis”) usually results from the traumatic inoculation on the skin or mucous membrane of environmental Sporothrix species present in soil and contaminated plant material (3,11). The zoonotic transmission of sporotrichosis was described sporadically involving bites or accidents with snakes, birds, mosquito, rat, horse, squirrel, fish, armadillo, and cats (13).

Clinically, most patients exhibit the lymphocutaneous form of sporotrichosis. It is initially characterized by a papulonodular lesion that appears few weeks after the trauma in the site of fungus inoculation, becoming ulcerated, draining a purulent discharge; later, the nodules usually progress along the regional lymphatic channels, and may ulcerate, fistulize, and heal. Other cutaneous manifestations include fixed cutaneous form usually as single ulcerative nodule with no regional lymphatic spreading; multiple inoculation form, usually in immunocompetent individuals with history of multiple traumas and disseminated skin lesions of polymorphic appearance, with no systemic invasion; and cutaneous disseminated form as systemic involvement appearing as larger well-defined ulcers or erythematous, papulopustular, vegetative, and crusty lesions. The disseminated sporotrichosis may affect the skin, bone, meninges, as well as oral, nasal, and conjunctival mucosa (6). The involvement of ocular mucosae is common, manifesting as conjunctivitis, episcleritis, uveitis, choroiditis, dacryocystitis, and retrobulbar lesions (3). The oral mucosa may be occasionally affected by sporotrichosis as multiple reddish to yellowish papules and ulcers of varying size and symptoms, mainly in the palate, tongue, buccal mucosa, as observed in the present cases (7,14). Bones and joints, the lungs and central nervous system may also be affected, particularly in immunocompromised patients. Systemic manifestations include hematogenous disseminated mucocutaneous lesions of the centrofacial region, severe bone lesions, lung and spleen involvement, neurotropism, and eventual progression to sepsis, leading to death. Some patients may heal spontaneously while others develop an exacerbated immune response against the fungus as hypersensitivity clinical forms, such as erythema nodosum, erythema multiforme, sweet’s syndrome, and polyarticular and migratory reactive arthritis that frequently disappears after the sporotrichosis treatment (3).

Due to the diversity of clinical presentations, sporotrichosis may be clinically similar to many other infectious and non-infectious diseases, both tegumentary and systemic. The most common are tegumentary leishmaniasis, pyodermitis, cat-scratch disease, cutaneous nocardiosis, chromomycosis, syphilis, rosacea, granuloma annulare, pyoderma gangrenosum, osteomyelitis, arthritis with a different etiology, such as rheumatoid, also cutaneous and pulmonary tuberculosis, tumoral lesions, especially in the lungs and in the central nervous system, and meningitis. The differential diagnosis of oral sporotrichosis include other infectious disease associated to immunosuppression that may exhibits oral manifestation such as cytomegalovirus (CMV) infection, tuberculosis, paracoccidioidomycosis, cryptococcosis and coccidiodomycosis. Despite the clinical aspects, the microscopical features of sporotrichosis are unspecific and the identification of *Sporothrix spp.* may be highlighted by PAS or silver-based stains. The presence of asteroid bodies within microabscesses is not pathognomonic but seems to be helpful in making a presumptive histologic diagnosis of sporotrichosis. The gold standard method is the isolation and the fungus identification through culture (6), as performed on the present cases.

Sporothrix species differ in their geographical distribution (10). Sporotrichosis has been reported as hyperendemic in some regions from Mexico (the Jalisco and Puebla Mountain with 25 cases per 1000 inhabitants), Peru (48 to 98 cases per 100,000 persons) and Japan (about 155 cases were reported every year from 1946 to 1982, with a recent decreasing to 50 cases per year), being *S. schenckii* sensu stricto and S. globosa the most common identified species (10). In Africa, India and Italy, human infection had also been attributed to another species, S. luriei (10). More than 5000 cases of sporotrichosis caused by S. brasiliensis were diagnosed in Rio de Janeiro from 1997 to 2015 (4,13). Since 2013, mandatory notification of sporotrichosis is required in the state of Rio de Janeiro, and there were approximately 3,300 cases between 2013 and 2016, with a median of 782 cases per year (4). The Evandro Chagas Clinical Research Institute reported 759 humans, 1503 cat, and 64 dog cases of sporotrichosis between 1998 and 2004, while Barros *et al*. described approximately 2200 human and 3244 feline cases diagnosed between 1998 and 2009, which is considered one of the largest cohorts of human and animal sporotrichosis in the world (10,16). Most affected patients are vulnerable children, elderly, and women of poor socioeconomic conditions that have frequent contact with animals, or have been infected by the HIV, or live in the municipalities of low human development index (16). The association of AIDS and disseminated forms of sporotrichosis is well known (8), suggesting that immunosuppression plays a role in the hematogenous dissemination of the fungus (1,16). The present patients showed the disseminated form of sporotrichosis in the setting of immunosuppression (tuberculosis and AIDS, respectively), one of them reporting that her cat have died with similar lesions, strongly suggesting the occurrence of S. brasiliensis zoonotic transmission. Both patients from the present study lives in the city of Rio de Janeiro, where the high prevalence of cases has created a sporotrichosis belt in this region, particularly represented by women adults from poor socioeconomic backgrounds with domiciliary or professional contact (bite or scratch) with cats infected with sporotrichosis (3,10).

In conclusion, we presented the clinicopathological features of two immunocompromised patients from Rio de Janeiro-Brazil with cutaneous disseminated form of sporotrichosis exhibiting oral manifestation, as result of zoonotic transmission of S. brasiliensis. The diagnosis of sporotrichosis is based on the correlation of clinical, microscopical and culture findings, including history of frequent contact with animals. In addition to other deep fungal infections, sporotrichosis should be considered in the differential diagnosis of multiple cutaneous and oral ulcers affecting vulnerable and immunocompromised patients from poor socioeconomic regions.
